# Merkel Cell Carcinoma Brain Metastasis Mimicking Stroke: A Case Report

**DOI:** 10.7759/cureus.70957

**Published:** 2024-10-06

**Authors:** Hiba Y Yaghmour, Rahaf O Al-Dwaik, Rahaf I AbuRayyan, Orwa Z Al-Fallah, Ibrahim Alzatari

**Affiliations:** 1 Medicine, Palestine Polytechnic University, Hebron, PSE; 2 Radiology, Palestine Polytechnic University, Hebron, PSE; 3 Radiology, Al-Ahli Hospital, Hebron, PSE

**Keywords:** brain metastasis, carcinoma, merkel cell cancer, neuroendocrine carcinoma, skin cancer

## Abstract

Merkel cell carcinoma (MMC) is a rare cutaneous neuroendocrine cancer that has the potential to metastasize. However, brain metastasis is infrequent in this type of cancer. We presented a case of a 55-year-old female patient with MCC who had a skin lesion on her right thigh. The cancer had spread to the brain and caused the development of splenic nodules. The patient also experienced intractable headaches and right-sided body weakness, which mimicked the symptoms seen in stroke cases. This case highlights the unusual clinical features and emphasizes the need for further investigation and understanding of the behavior of MCC, particularly in relation to metastasis patterns and associated symptoms.

## Introduction

Merkel cell carcinoma (MCC) is a rare aggressive rapidly progressive neuroendocrine cancer of the skin [1]. It was first described as trabecular carcinoma of the dermis by Cyril Toker in 1972 [1]. The annual incidence of MCC globally is estimated to be approximately 0.13-1.6 cases per 100000 individuals, and this incidence is increasing over time with higher occurrence in males [1]. In addition to its rapid local growth, MCC is characterized by a fast progression at the systemic level [2]. MCC commonly spreads to the lymph nodes and distant organs, such as the liver, bone, pancreas, lung, and brain [2]. Given that MCC tends to affect elderly individuals and exhibits a high potential for metastasis, the prognosis for MCC is poor [2]. We presented a case of a patient with brain metastasis with splenic nodules arising from MCC. 

## Case presentation

**Figure 1:**
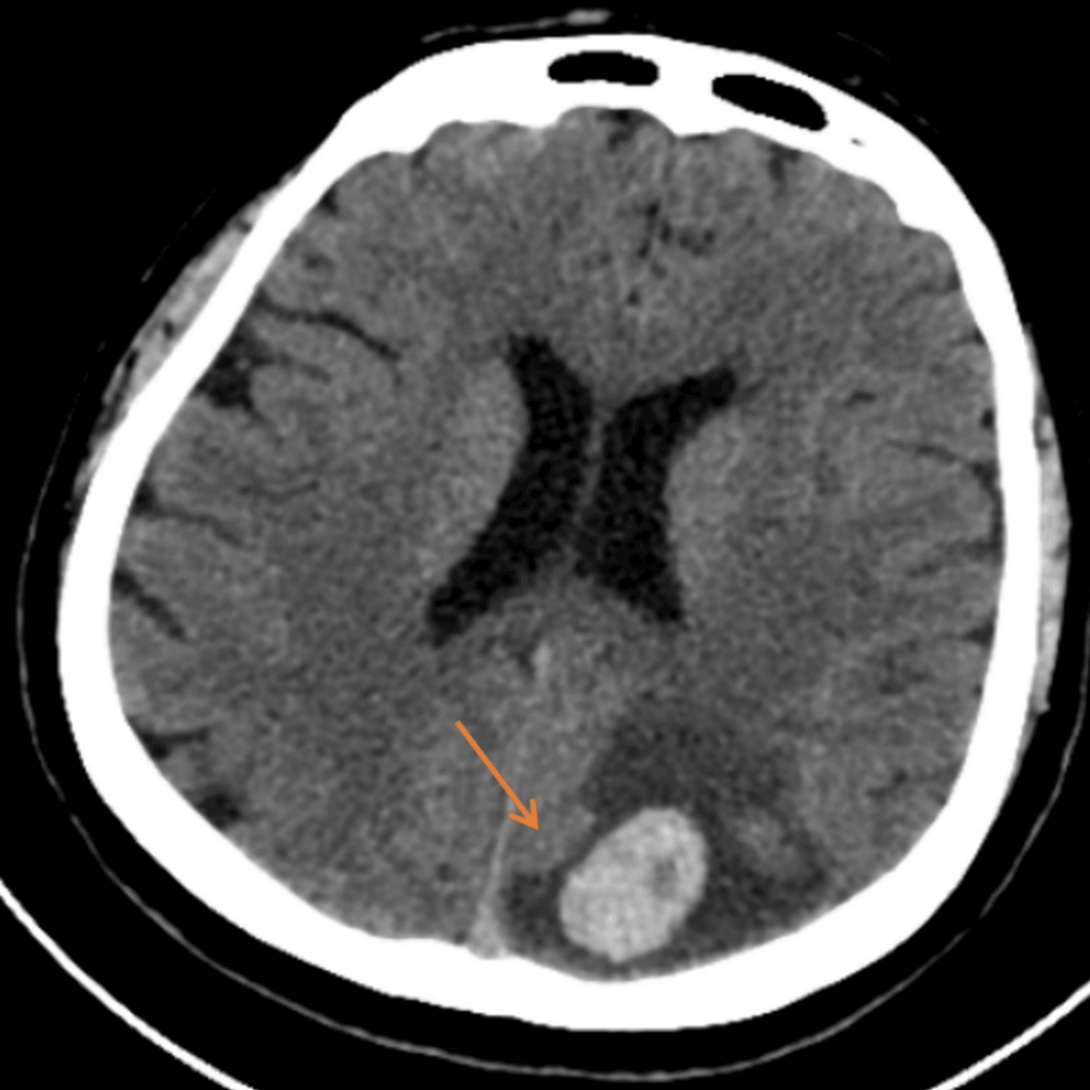
Initial non-enhanced brain CT The axial plane showed a hyperdense lesion at the left parietal lobe surrounded by vasogenic edema with mild effacement of adjacent sulci. Conclusion of the first CT: This was assumed to be an intraparenchymal hemorrhage or hemorrhagic lesion such as a ruptured cavernoma.

**Figure 2:**
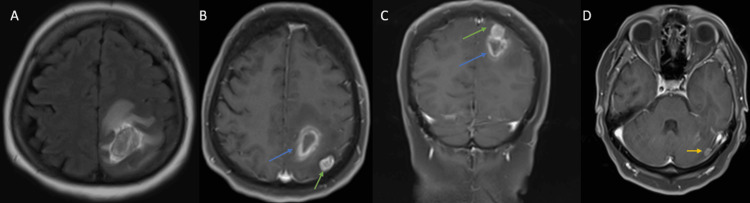
Brain MRI without and with IV contrast A: Axial flair; B, C, and D: coronal and axial T1 post-contrast images. There is the peripheral enhancement of the mentioned lesion (blue arrow); however, another nodular-enhancing lesion is seen post-contrast. One is located adjacent to the former in the left parietal lobe (green arrow), in addition to a smaller one in the left cerebellar hemisphere (yellow arrow).

A 55-year-old female presented to our neurological department in August 2023 as a referral case of suspected intracerebral hemorrhage. She was complaining of right-sided body weakness associated with stiffness and headache for one week. She had a history of herpes zoster. Physical examination showed enlarged palpable right inguinal lymph nodes with a painless round cutaneous\subcutaneous lesion seen at the posterolateral aspect of the right upper thigh, measuring about 3 cm in diameter. Lab investigations were done but unremarkable (Table 1). Brain CT was done to confirm the primary diagnosis of a hemorrhagic lesion (Figure 1). Multiplanar/multisequence brain MRI without and with contrast was done that showed multiple enhancing lesions, the largest one is hemorrhagic. The most consistent diagnosis according to the findings is cerebral metastases (Figure 2).

**Table 1:** Lab investigations

Test	Result	Normal range (unit)
Complete blood count		
White blood cells	7.825 × 10^3^/µL	(5-10) × 10^3^/µL
Red blood cells	4.438 × 10^6^/µL	4.2-5.4 × 10^6^/µL
Hemoglobin	13.78 g/dL	11-15 g/dL
Mean corpuscular volume	93.554 fL	82-92 fL
Lymphocytes	21.9%	25%-45%
Eosinophil	7.84%	0-2%
Tumor markers		
Cancer antigen 19-9	<2 U/mL	0-37 U/mL
Cancer antigen 125	11 U/mL	0-35 U/mL
Carcinoembryonic antigen	0.55 ng/mL	Nonsmokers: up to 3 ng/mL; smokers: up to 5 ng/mL
Hematology		
Erythrocyte sedimentation rate	25 mm/hour	0-20 mm/hour
Chemistry		
Serum glutamic pyruvic transaminase	62 U/L	0-45 U/L
Serum glutamic oxaloacetic transaminase	62 U/L	0-31 U/L

**Figure 3:**
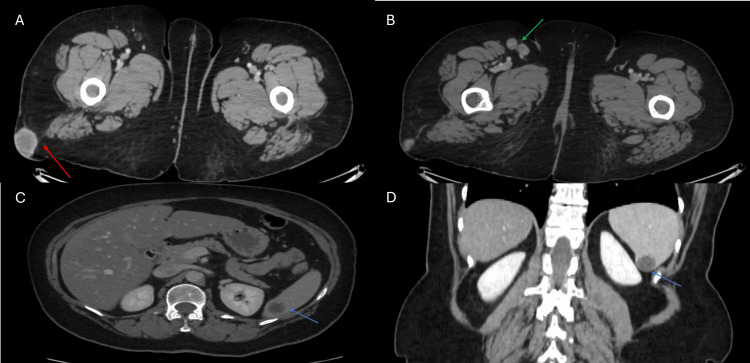
Abdomen and pelvis CT (A, B) Axial and coronal CT images with IV contrast (venous phase): At the lower cuts of the abdomen and pelvis, a CT scan was performed as part of the workup. There is a suspicious enhancing soft tissue lesion in the right subcutaneous gluteal region/proximal thigh, measuring 3 cm (red arrow). Also, noted are two round pathological-looking right inguinal lymph nodes, the largest measuring 1.8 cm x 1.4 cm (green arrow). C, D: A well-defined hypodense lesion showing near fluid density seen at the lower pole of the spleen (blue arrow).

Based on physical examination and imaging investigations, an abdominal and pelvic CT scan with IV contrast was performed. During the scan, a probable painful, round, firm nodular, and red-colored cutaneous/subcutaneous lesion was found in the right thigh, along with enlarged right inguinal lymph nodes (Figure 3). Additionally, a well-defined hypodense splenic lesion with a density resembling fluid was visualized in the lower pole, measuring approximately 1.5 cm (Figure 3).

**Figure 4:**
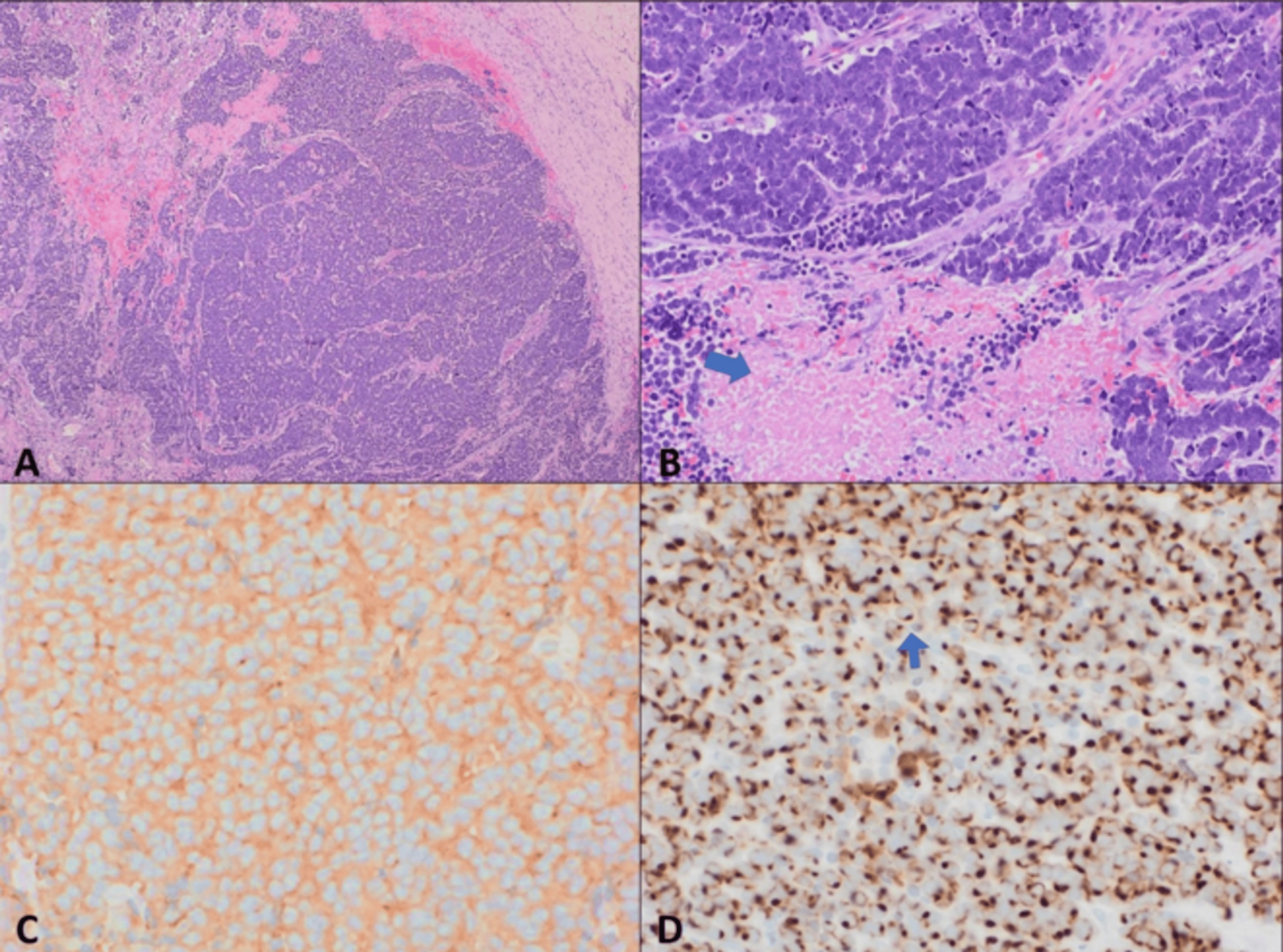
Biopsy samples A: The section revealed a dermal-based tumor composed of nodules of undifferentiated small round basophilic cells (H&E, 4X). B: The tumor cells are uniform, with a high nuclear-to-cytoplasmic ratio, finely stippled chromatin (salt-and-pepper pattern), high mitotic activity, and foci of necrosis (arrow) (H&E, 20X). C: Synaptophysin immunostain: diffusely positive. D: CK20 immunostain: positive (dot-like pattern).

An excisional biopsy of the skin measuring 5 cm x 4 cm x 3cm was taken. Step sectioning revealed the presence of a 3.2 cm × 2.2 cm nodular necrotic cutaneous tumor. A dermal-based tumor consisting of nodules of undifferentiated small, round basophilic cells was discovered under a microscope. The tumor cells have high mitotic activity, patches of tumor cell necrosis, a high nuclear-to-cytoplasmic ratio, finely stippled chromatin (salt and pepper pattern), and exhibit uniformity. Immunohistochemical findings for synaptophysin and cytokeratin 20 (CK20) were positive, while negative results for cytokeratin 7, S100, desmin, and GATA3 immunostains. The KI67 proliferation index was approximately 70% (Figure 4). Furthermore, one of the two lymph nodes displayed evidence of metastasis, with a deposit measuring 2 cm x 1.2 cm and presenting with extranodal extension.

Therefore, according to the history, physical examinations, lab results, imaging investigations, and biopsy findings the diagnosis of MCC was confirmed. So she was referred to another hospital to receive radiotherapy treatment immediately, but based on the rapid deterioration of her medical status doctors decided to keep her on palliative treatment.

## Discussion

The presented case reports multiple metastases in the body, with a focus on the intracranial region, which causes symptoms that mimic stroke. Furthermore, during the evaluation of the tumor, splenic nodules were discovered, which originated from a skin lesion in the posterolateral aspect of the right thigh. Based on the gathered information, there are only 31 reported cases of brain metastasis documented in the literature [3]. Furthermore, 98% of MCC patient cases are Caucasian, and the first case was discovered in a Panamanian patient [4].

MCC is an infrequent and highly aggressive form of cutaneous neuroendocrine malignant cancer [1]. It primarily emerges within the basal layer of the epidermis and can also be found in hair follicles, often proximal to nerve fibers [5].

The exact cause of MCC is still unclear, but a recent significant risk factor that has been discovered is a polyomavirus called Merkel cell polyomavirus. Other potential risk factors include advanced age, immunosuppression (as seen in organ transplant recipients, HIV-infected individuals, and those with B-cell malignancies), a history of sun exposure, and certain skin conditions such as squamous cell carcinoma [6,7]. Nevertheless, there is no proof indicating a relation between these risk factors and this patient. Herpes zoster virus was the only significant medical history, and no studies have established a definitive relationship between them.

Clinically, MCC typically presents as a rapidly growing, painless, firm nodule that may be red or flesh-colored with a smooth surface in elderly individuals [4]. Skin lesions are usually found in the head and neck (29%), followed by upper limbs (24%) and lower limbs (21%), and the rest of the cases are found in another site of the body or may not be seen in the skin in 4% of cases [3].

MCC tends to frequently spread through the lymphatic system, leading to regional and distant metastases [8]. These metastases could be involved in skin, lymph nodes, liver, bone, lung, and brain. In this case, the patient developed brain metastasis, which accounts for about 3%-5% of all metastatic reported cases [7]. The clinical symptoms vary between patients according to the metastatic site. By way of illustration, brain metastasis causes increased intracranial pressure that leads to the development of variable symptoms, including headaches, confusion, dizziness, visual disturbances, weakness, paralysis, or paresthesia, as well as nausea and vomiting [4].

According to the American Joint Committee on Cancer consensus guidelines, MCC has five stages: Stage 1 primary tumor ≤2 cm maximum tumor dimension; Stage 2 A >2 cm tumor dimension; Stage 3 any size tumor with invasion; Stage 4 any tumor size with metastasis [9]. Based on this criterion of staging, a survival rate of 81%, 67%, 52%, and 11%, respectively [3].

The skin lesion's appearance can be confused with benign lesions like lipomas or epidermal cysts, dermatofibroma, as well as malignant lesions such as basal cell carcinoma and melanoma, squamous cell carcinoma, lymphoma, sarcoma, MCC, and metastatic carcinoma [1].

Identification of the characteristic features depends on the biopsy of the suspicious lesion that was examined under a microscope. A typical pattern of MCC cells often has a pepper and salt appearance, high mitotic activity, a high nuclear-to-cytoplasmic ratio, and patches of necrosis [10].

All appeared on this patient’s biopsy result. Furthermore, the immunohistochemical analyses provided additional diagnostic support. Generally, neuroendocrine markers: chromogranin-A, synaptophysin, and CK20, are present in MCC cells [5]. The last one has the highest specificity for MCC and can distinguish it from other neuroendocrine tumors [6]. In this case, six immunostatins were tested: CK7, S100, desmin, GATA3, synaptophysin, and CK20. The first four were negative, which excluded the other differential diagnoses, but the only two positives were CK20 and synaptophysin, which confirmed the diagnosis of MCC. CK20 staining has high specificity for MCC and can distinguish it from other neuroendocrine tumors [6].

The atypical late presentation of this patient with no specific risk factors for MCC makes it challenging to diagnose and treat. Surgery, chemotherapy, and radiotherapy are the options that are typically used to treat MCC, which are determined according to the patient's history and the stage of cancer [1]. This patient was referred to an oncologist for treatment and follow-up.

## Conclusions

In conclusion, this case illustrates an atypical presentation of a 55-year-old female patient diagnosed with MCC. MCC is a rare and aggressive neuroendocrine tumor capable of metastasis. She exhibited unusual clinical characteristics, with no recognized risk factors connected with this tumor. She had brain metastasis, which is uncommon in this carcinoma with splenic nodules. Moreover, the patient had neurological symptoms mimicking stroke, underscoring the unusual presentation. Her case highlights the need for understanding MCC and puts it at the top of differential diagnosis in a similar presentation because early detection and a multidisciplinary treatment approach are crucial for improving outcomes in MCC.
